# Fatal Acute Thyroiditis in a Giraffe (*Giraffa camelopardalis*) Associated with *Clostridium perfringens* Type A: A “Local Proliferation–Systemic Intoxication” Pathogenic Model

**DOI:** 10.3390/ani16132006

**Published:** 2026-07-01

**Authors:** Guoxin Hao, Zhixin Fu, Jing Li, Shengxin Zeng, Jingjing Hu, Yongbo Liu

**Affiliations:** 1 Hebei Key Laboratory of Preventive Veterinary Medicine, College of Animal Science and Technology, Hebei Normal University of Science & Technology, Qinhuangdao 066000, China; 2 Clinical Research and Innovation Center for Ruminant Animals, College of Animal Science and Technology, Hebei Normal University of Science & Technology, Qinhuangdao 066000, China; 3 College of Veterinary Medicine, Sichuan Agricultural University, Chengdu 611130, China

**Keywords:** giraffe, *Clostridium perfringens* type A, acute thyroiditis, α-toxin, pathogenic model

## Abstract

This report presents the first case of fatal acute thyroiditis associated with *Clostridium perfringens* type A in a captive giraffe (*Giraffa camelopardalis*) and describes findings consistent with the proposed “local proliferation–systemic intoxication” pathogenic model. A 5-year-old female giraffe in Hebei Province, China, presented with diarrhea, emaciation, and sudden death. Necropsy revealed marked thyroid enlargement, intestinal mucosal hemorrhage and shedding, and pericardial effusion. *C. perfringens* type A was isolated from thyroid tissue, and molecular analysis detected the presence of the α-toxin gene (*cpa*). Immunohistochemistry detected α-toxin antigen immunoreactivity in thyroid, lung, liver, and cardiac tissues. Histopathological examination showed severe disruption of thyroid follicular structures and inflammatory cell infiltration, alongside α-toxin immunoreactivity in multiple organs, including the lungs, liver, and heart. Based on the observed pathological and microbiological findings, we hypothesize a possible pathogenic scenario in which intestinal barrier disruption may facilitate bacterial dissemination and subsequent thyroid colonization. This report expands the known clinical spectrum of *C. perfringens* infections and provides insights into possible atypical clostridial pathogenic mechanisms in non-classical host species.

## Simple Summary

Health problems in captive wildlife are often hard to detect early, especially when clinical signs are nonspecific, leading to delayed diagnosis. In this case, a female giraffe initially showed gastrointestinal discomfort and subsequently deteriorated rapidly and died. Postmortem examination revealed severe inflammatory damage to the thyroid gland, an extremely rare lesion in giraffes. Laboratory analyses led to the isolation of *Clostridium perfringens* type A from the affected tissue, suggesting a possible association between this bacterium and extraintestinal lesions. This case offers valuable insights. First, it broadens the differential diagnosis of sudden death in large herbivores. Second, it suggests that abnormal organ enlargement may not indicate an isolated local lesion but rather a manifestation of systemic toxin exposure. Recognizing such atypical presentations can improve clinical diagnosis, treatment, and disease control in zoo and wildlife medicine. It also reminds practitioners that common pathogens can sometimes cause atypical and critical illnesses.

## 1. Introduction

Giraffes are widely maintained in zoological collections worldwide. Although infectious diseases have been reported in captive giraffes, information regarding *Clostridium perfringens* (*C. perfringens*)-associated disease in this species remains limited. Among these pathogens, *C. perfringens* is an important opportunistic zoonotic pathogen and a well-recognized cause of enteric disease and toxin-mediated tissue injury [1,2,3,4]. Type A strains are the most prevalent toxinotype and have been associated with enteritis, enterotoxemia, and sudden death in various animal species [5,6,7,8,9]. Although the pathological manifestations of *C. perfringens* infection have been extensively documented, the lesions primarily involve the gastrointestinal tract, whereas reports of extraintestinal lesions are far less common [4,7].

Although infections caused by *C. perfringens* type A have been widely reported [6,10], extraintestinal involvement remains relatively rare. In particular, the clinical manifestations of this pathogen in giraffes, especially lesions associated with thyroid pathology, have not yet been reported. The thyroid gland is a vital endocrine organ in animals, and the integrity of its structure and function directly affects key physiological processes such as metabolism, growth and development, and immune regulation [11]. Thyroid enlargement has traditionally been associated with iodine deficiency or autoimmune diseases [12,13]. In contrast, acute thyroiditis associated with bacterial infection, especially involving enteric pathogens such as *C. perfringens*, represents a rare and atypical clinical manifestation.

We searched the PubMed, Web of Science, Scopus, and Google Scholar databases for literature published up to January 2026, using search terms including “*Clostridium perfringens*”, “giraffe”, “*Giraffa camelopardalis*”, “thyroiditis”, “thyroid colonization”, and combinations thereof. No reports describing *C. perfringens*-associated thyroiditis in giraffes were found. Therefore, to the best of our knowledge, this is the first documented case of *C. perfringens*-associated thyroiditis in a giraffe. In this report, we describe the clinical, pathological, bacteriological, and immunohistochemical findings of a giraffe with *C. perfringens*-associated thyroiditis and discuss the possible association between thyroid involvement and systemic toxin distribution.

## 2. Case Description

### 2.1. Animal Management

The giraffe was housed in a mixed-species wildlife park enclosure and was fed a diet consisting primarily of alfalfa hay, fresh browse, and commercial pelleted feed formulated for giraffes. According to veterinary records, no major medical conditions had been reported previously. Routine deworming had been performed, and no recent vaccination-related adverse events were recorded. During the autumn–winter transition, marked fluctuations in ambient temperature were observed, which may have represented a potential environmental stressor.

### 2.2. Clinical Presentation

In November 2025, a 5-year-old female giraffe weighing 550 kg at a wildlife park in Hebei Province presented with the clinical course detailed in Table 1. Briefly, diarrhea and weight loss began on day–5. Deworming on day–4 did not improve the condition. Diarrhea persisted until day–1. On the morning of day 0, acute abdominal distension developed, and the animal died at 6:00 p.m. that day. No similar gastrointestinal signs or sudden deaths were reported in other giraffes or animal species housed in the same wildlife park during the same period. Therefore, this case was considered an isolated event rather than part of a recognized outbreak.

### 2.3. Necropsy Findings

**Figure 1:**
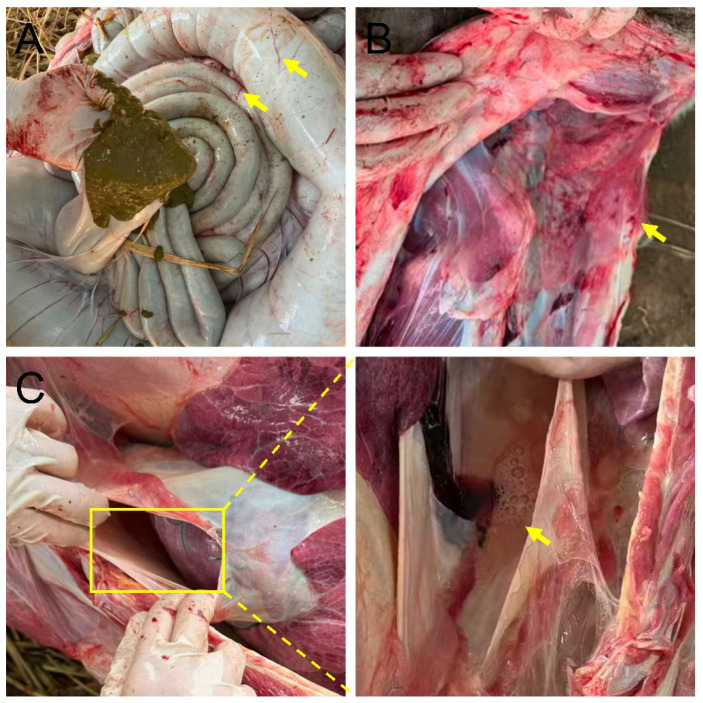
Gross necropsy findings. (**A**) The small intestine (jejunum-ileum region) shows diffuse petechial-to-miliary mucosal hemorrhages (yellow arrows). (**B**) Enlargement of the thyroid gland with a smooth external surface (yellow arrows). (**C**) Heart showing marked accumulation of pale-yellow pericardial effusion within the pericardial cavity (yellow arrows); the lower-right inset, also part of Figure 1C, further illustrates this finding (yellow arrows).

Necropsy performed within 1 h postmortem revealed significant multisystem pathological changes. The mucosa of the rumen and reticulum showed extensive patchy shedding with loss of normal architecture. The small intestine contained a large volume of watery fluid, and the mucosal surface exhibited diffuse petechial-to-miliary hemorrhages (Figure 1A). Additionally, both lobes of the thyroid gland were markedly enlarged with a smooth surface (Figure 1B); although precise measurements were not recorded at necropsy, the thyroid was grossly enlarged compared to the expected size for a giraffe of similar age. The pericardial cavity contained more than 1 L of clear, pale-yellow pericardial effusion (Figure 1C), accompanied by auricular injury manifested as swelling and softening of the tissue.

### 2.4. Laboratory Examination

**Figure 2:**
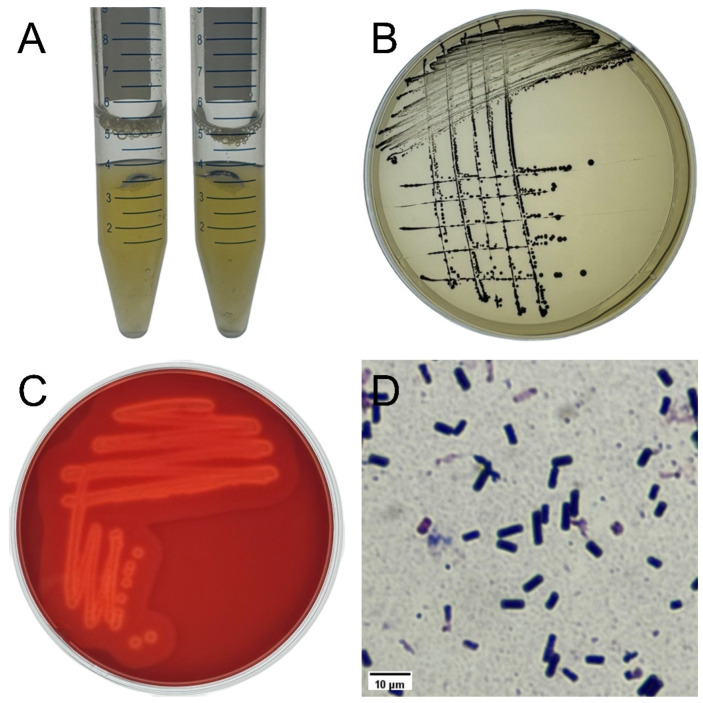
Cultural characteristics and staining microscopy of the isolated strain. (**A**) RCM medium. (**B**) TSC medium. (**C**) 7% sheep blood agar medium. (**D**) Morphology of the isolated bacteria under microscopy (1000×; 10× ocular, 100× objective).

Based on the clinical presentation and necropsy findings, clostridial infection was considered among the differential diagnoses, and microbiological investigation was therefore undertaken [14,15]. To isolate the suspected pathogen, a specific isolation and cultivation method for *C. perfringens* was employed. The specific procedures were as follows: A thyroid tissue sample (approximately 100 mg) was aseptically collected, homogenized, and inoculated into 10 mL of Reinforced Clostridial Medium (RCM, Solarbio, Beijing, China). The sample was then anaerobically incubated at 37 °C for 24 h. During anaerobic incubation, visible turbidity and gas production were observed in the liquid medium (Figure 2A). The positive culture was subsequently streaked onto Tryptose–Sulfite–Cycloserine (TSC) agar and incubated anaerobically at 37 °C for 24 h. Black, circular colonies (2–5 mm in diameter) surrounded by a characteristic opaque halo appeared on the plates (Figure 2B). A typical black, circular, single colony was picked and transferred to RCM medium for further purification and incubation for another 24 h. Finally, the purified bacterial suspension was streaked onto 7% sheep blood agar plates. After incubation at 37 °C, smooth, grayish-white, translucent circular colonies were formed (Figure 2C). Grayish-white single colonies surrounded by a hemolytic ring were selected for Gram staining and microscopic examination, which revealed Gram-positive short bacilli (Figure 2D).

**Figure 3:**
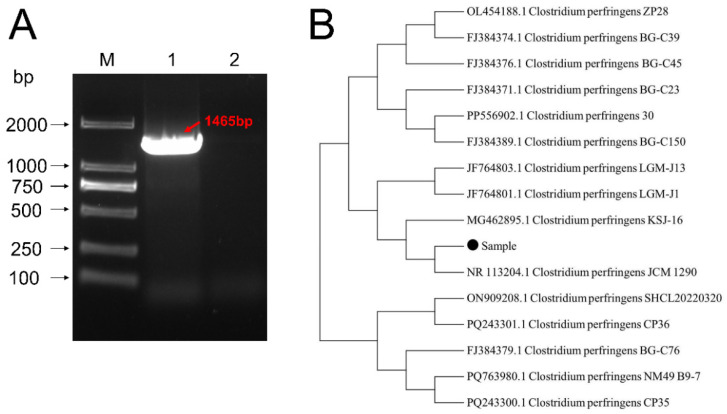
Molecular identification results of the isolated strain. (**A**) Agarose gel electrophoresis of PCR-amplified 16S rRNA gene products. M: DNA marker; 1: amplified product from the isolate (1465 bp); 2: negative control. (**B**) Phylogenetic tree based on 16S rRNA gene sequences.

To further determine the taxonomic status of the strain, the total genomic DNA of the isolate was extracted using the FastPure Viral DNA/RNA Extraction Kit (Vazyme Biotech Co., Ltd., Nanjing, China). The extracted DNA was used as a template for polymerase chain reaction (PCR) amplification with universal 16S rRNA primers (see Table 2). Nuclease-free water was set as a negative control in each PCR. Samples yielding an amplicon of the expected size were determined to be PCR-positive. The total PCR reaction volume was 50 μL, consisting of 25 μL of 2× Green Taq Mix, 1 μL each of the forward and reverse primers, and 2 μL of DNA template, with the remaining volume made up to 50 μL with double-distilled water (ddH_2_O). The amplification program was set as follows: initial denaturation at 94 °C for 4 min; followed by 30 cycles of denaturation at 94 °C for 30 s, annealing at 57 °C for 30 s, and extension at 72 °C for 90 s; and a final extension at 72 °C for 10 min, with subsequent storage at 4 °C. The PCR products were verified by 1.5% agarose gel electrophoresis and showed an amplified band of approximately 1465 bp, consistent with the expected size (Figure 3A). The amplified products were sent to Tsingke Biotechnology Co., Ltd. (Beijing, China) for sequencing. The 16S rRNA gene sequence of the isolate has been deposited in GenBank under accession number PZ506140. BLAST analysis (https://blast.ncbi.nlm.nih.gov/Blast.cgi, accessed on 28 June 2026) in the NCBI database, combined with phylogenetic tree construction using MEGA 11.0.13 software, revealed that the isolate shared sequence similarities of over 99% with reference strains of *C. perfringens* and clustered within the same branch as a *C. perfringens* sequence (NR113204.1) submitted from Japan (Figure 3B), indicating a close genetic relationship. Based on these analyses, the isolate was identified as *C. perfringens*.

**Figure 4:**
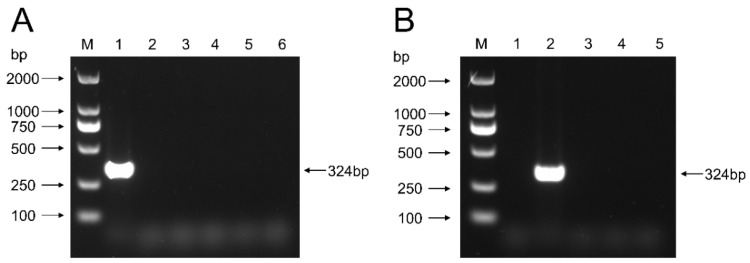
PCR-based genotyping and tissue distribution of *C. perfringens* toxin genes. (**A**) Electrophoretic analysis of toxin gene genotypes. M. DNA marker; 1. *cpa* gene; 2. *cpb* gene; 3. *etx* gene; 4. *itx* gene; 5. *netB* gene; 6. *cpe* gene. (**B**) Detection of *cpa* gene distribution in tissues. M.DNA marker; 1. liver; 2. thyroid; 3. lung; 4. heart; 5. negative control.

To determine the toxin genotype of the isolated strain, toxin gene typing was performed by PCR using purified bacterial culture as the template, and toxin gene distribution in different tissues was analyzed. The toxin genotyping PCR had a total volume of 20 μL, containing 2 μL of template, 1 μL each of the forward and reverse primers, 10 μL of 2× Green Taq Mix, and the remaining volume supplemented with ddH_2_O to 20 μL. Each primer pair was set up in a separate reaction system. The toxin genes were amplified using primer sequences reported in the literature [16] (Table 2). A negative control was set up in each assay. Detection of a specific amplification product of the expected size was interpreted as a positive result. The reaction conditions were as follows: 95 °C for 3 min for initial denaturation; followed by 35 cycles (95 °C for 30 s, 55 °C for 30 s, and 72 °C for 30 s); and a final extension at 72 °C for 10 min. PCR results showed that the isolated strain yielded a specific band of the expected size (324 bp) only at the *cpa* gene locus. No bands were detected for the other toxin genes, indicating that the strain is type A *C. perfringens* (Figure 4A).

For the detection of *cpa* gene distribution in tissues, genomic DNA was extracted from various lesioned tissues using the FastPure Viral DNA/RNA Extraction Kit. The concentration and purity of the DNA were measured with a NanoDrop 2000 spectrophotometer (Thermo Fisher Scientific, Waltham, MA, USA). Subsequently, tissue DNA was used as the template, and the PCR system and program referenced the toxin genotyping protocol described above, with primers listed in Table 2. Tissue-specific PCR analysis showed that the *cpa* gene was detected only in thyroid tissue and was not detected in the liver, heart, or lung tissues (Figure 4B).

**Table 2:** Sequences of primer pairs used for amplification of target and reference genes.

Genes	Primer Sequences (5′-3′)	Product Length (bp)
16S rRNA	F-AGAGTTTGATCCTGGCTCAG	1465 [17]
	R-GGTTACCTTGTTACGACTT	
*cpa*	F-GCTAATGTTACTGCCGTTGA	324
	R-CCTCTGATACATCGTGTAAG	
*cpb*	F-GCGAATATGCTGAATCATCTA	196
	R-GCAGGAACATTAGTATATCTTC	
*etx*	F-GCGGTGATATCCATCTATTC	655
	R-CCACTTACTTGTCCTACTAAC	
*itx*	F-ACTACTCTCAGACAAGACAG	446
	R-CTTTCCTTCTATTACTATACG	
*netB*	F-GCTGGTGCTGGAATAAATGC	384
	R-TCGCCATTGAGTAGTTTCCC	
*cpe*	F-GGAGATGGTTGGATATTAGG	233
	R-GGACCAGCAGTTGTAGATA	

**Figure 5:**
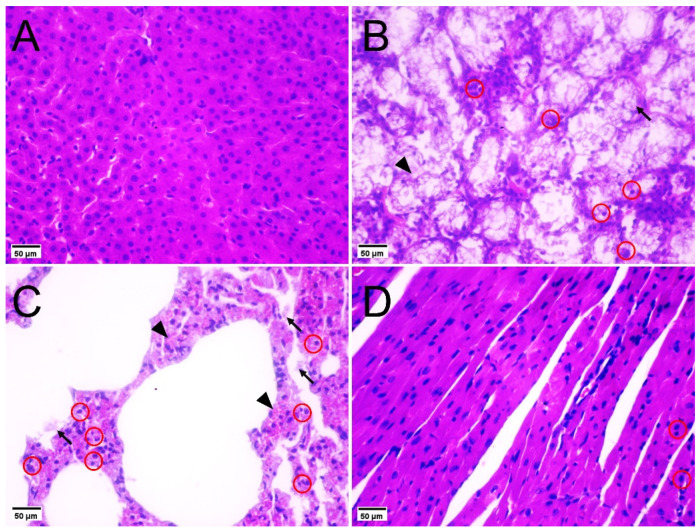
Histopathological examination. (H&E) (**A**) Liver tissue showing no significant histological abnormalities. (**B**) Thyroid gland exhibiting disorganized to absent follicular structures (arrowheads), accompanied by focal mild inflammatory cell infiltration (red circles). Abundant filamentous proteinaceous exudates are visible within the follicular lumens (arrows). (**C**) Lung tissue displaying extensive hemorrhagic foci (arrowheads) and inflammatory cell infiltration (red circles); proteinaceous exudates are observed within the alveolar spaces (arrows). (**D**) Myocardial tissue showing scattered sparse inflammatory cells (red circles), with no significant pathological changes identified. Scale bar = 50 μm. Magnification: 400× for all panels.

Liver, thyroid, lung, and heart tissue samples were processed according to standard histological protocols. After fixation in 10% neutral buffered formalin for 24 h, they were sequentially dehydrated through a graded ethanol series, cleared in xylene, and infiltrated with paraffin. Finally, the samples were embedded to produce serial sections with a thickness of 5 μm. The sections were deparaffinized in xylene and rehydrated through a descending ethanol gradient before being stained with hematoxylin and eosin (H&E) following standard procedures. After dehydration and clearing, the slides were mounted with neutral balsam [18]. The H&E-stained sections were examined under an optical microscope (EX30) (SOPTOP Instrument Co., Ltd., Yuyao, China). Histopathological analysis revealed the following changes. The liver tissue showed no significant abnormalities, with no observable inflammatory cell infiltration or edema (Figure 5A). The thyroid tissue exhibited disorganized or even absent follicular structures, along with mild inflammatory cell infiltration and abundant filamentous proteinaceous exudates within the follicular lumens (Figure 5B). The lung tissue demonstrated extensive hemorrhage and inflammatory cell infiltration, accompanied by thickening of the interstitium, disruption of alveolar architecture, and the presence of proteinaceous exudates and sloughed cells within the alveolar spaces (Figure 5C). In the cardiac tissue, myocardial fibers were orderly arranged, with no hemorrhagic lesions observed; only occasional minimal inflammatory cell infiltration was noted, and no significant pathological changes were identified (Figure 5D).

Immunohistochemical staining was performed using the streptavidin–biotin–peroxidase complex (SP) method. The main steps were as follows: Tissues were fixed for more than 48 h, rinsed under running water for 20 min, dehydrated through a graded ethanol series, cleared in xylene, and embedded in paraffin. Sections were cut at 4 μm thickness, deparaffinized in xylene, and rehydrated through a descending ethanol gradient. Antigen retrieval was performed by incubating sections with trypsin (1:100 in PBS) at 37 °C for 20 min. Endogenous peroxidase activity was blocked with 3% H_2_O_2_ for 15 min at room temperature in the dark. Non-specific binding was blocked with 10% normal goat serum for 30 min at room temperature. Sections were incubated overnight at 4 °C with a rabbit polyclonal antiserum against purified *C. perfringens* type A α-toxin, serving as the primary antibody. This antiserum was prepared by immunizing rabbits with purified α-toxin derived from deer-origin *C. perfringens* type A. Positive controls consisted of mouse intestinal tissues treated with purified *C. perfringens* type A α-toxin, whereas negative controls consisted of mouse intestinal tissues without α-toxin treatment. Representative images of the positive and negative IHC controls are provided in Supplementary Figure S2. The antiserum exhibited an ELISA titer of 1:10,000 and was used at a working dilution of 1:400 for IHC. Subsequently, sections were incubated with an HRP-labeled secondary antibody for 50 min at room temperature. Immunoreactivity was visualized using DAB substrate, and sections were counterstained with hematoxylin. Finally, sections were dehydrated, cleared, and mounted with neutral balsam. Background staining was evaluated in non-target tissues, and IHC intensity was scored semi-quantitatively as weak, moderate, or strong.

**Figure 6:**
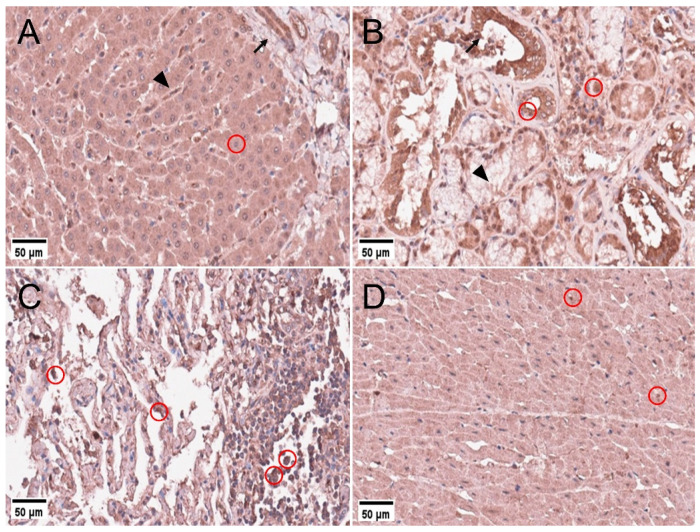
Immunohistochemical detection of α-toxin antigen immunoreactivity in multiple tissues. (**A**) Liver: Brown α-toxin antigen immunoreactivity is primarily localized to the cuboidal epithelial cells of the bile ducts (arrowheads) and the endothelial cells of the hepatic sinusoids (arrows). No obvious immunoreactivity is observed in the hepatocytes (red circles). (**B**) Thyroid: Marked nuclear dissolution and disappearance are observed in the follicular cuboidal epithelial cells (arrowheads), with necrotic changes in the follicular structure (arrows). Widespread brown α-toxin antigen immunoreactivity is detected in both follicular cells and parafollicular cells (red circles). (**C**) Lung: Extensive α-toxin antigen immunoreactivity is detected throughout the lung tissue (red circles). (**D**) Heart: Extensive α-toxin antigen immunoreactivity is detected throughout the cardiac muscle tissue (red circles). Scale bar = 50 μm. Magnification: 400× for all panels.

Immunohistochemical results showed that in the liver tissue, α-toxin antigen immunoreactivity was predominantly detected in the cuboidal epithelial cells of the bile ducts and the endothelial cells of the sinusoids, while no obvious immunoreactivity was observed in the hepatocytes (Figure 6A). In the thyroid tissue, a large number of follicular cuboidal epithelial cells exhibited nuclear dissolution and disappearance, accompanied by necrosis of the follicular structure, and widespread brown α-toxin antigen immunoreactivity was detected in both follicular cells and parafollicular cells (Figure 6B). In both lung tissue and cardiac muscle tissue, extensive α-toxin antigen immunoreactivity was detected (Figure 6C,D).

## 3. Discussion

As a common anaerobic commensal bacterium in the animal intestinal tract, *C. perfringens* plays a certain role in maintaining intestinal homeostasis under healthy conditions by participating in short-chain fatty acid metabolism and regulating intestinal mucosal anti-inflammatory responses [19,20]. Under pathological conditions such as intestinal mucosal injury, the integrity of the intestinal barrier is compromised. This leads to the translocation of commensal bacteria, including *C. perfringens*, which then invade the bloodstream or other sterile sites. Thus, they transform from commensals to pathogenic bacteria [19,21,22]. Additionally, *C. perfringens* exhibits seasonal epidemiological patterns, with a higher incidence in winter than in other seasons [23,24]. Previous studies have shown that cold stress can impair immune function and compromise intestinal barrier integrity, which may increase susceptibility to opportunistic infections. It may also contribute to intestinal dysmotility, maldigestion, malabsorption, and microbial dysbiosis [25]. This case occurred in November 2025, during the autumn–winter transition in the Northern Hemisphere, in northern China. Environmental and husbandry-related factors may have influenced host susceptibility to disease.

In this case, PCR detected the *cpa* gene exclusively in thyroid tissue, whereas immunohistochemistry (IHC) revealed widespread α-toxin antigen immunoreactivity in the examined tissues. This discrepancy between nucleic acid and protein detection is compatible with a possible “local proliferation–systemic intoxication” pathogenic model: the thyroid gland may have represented a localized site for bacterial replication and localized toxin generation, from which released α-toxin may have entered the circulation and distributed to distant organs, which may explain why toxin antigen, but not bacterial DNA, was detected in the heart, lung, and liver. Crucially, IHC detects α-toxin-associated epitopes rather than biologically active toxins; therefore, positive signals may represent the systemic deposition of circulating toxin antigens rather than definitive evidence of local tissue destruction. In the heart and liver, despite widespread α-toxin immunopositivity, no obvious histological damage was observed. One possible explanation is that this case represents a hyperacute intoxication process, in which the animal died rapidly after the toxin had already spread and bound to tissues, so that conventional histopathological examination did not yet reveal discernible structural lesions. Therefore, IHC findings should be regarded as supportive evidence of systemic toxemia rather than definitive proof. Other explanations for the PCR-IHC discrepancy include bacterial DNA load below the PCR detection limit, focal distribution of bacteria leading to sampling variation, DNA degradation, postmortem changes, or differences in analytical sensitivity between the two methods. None of these possibilities can be completely excluded. Furthermore, since microbiological cultures were not performed on intestinal contents, blood, spleen, or lymphoid tissues, bacterial involvement at these sites cannot be ruled out. These findings are compatible with the proposed model but should be interpreted cautiously.

Why was *C. perfringens* detected in the thyroid? There is no direct evidence that this bacterium has a specific tropism for thyroid tissue. Under normal conditions, the thyroid is highly vascularized with high oxygen partial pressure, making it unsuitable for the growth of obligate anaerobic bacteria [26]; however, in some cases, disruption of thyroid follicular architecture with necrosis and inflammatory exudation may have altered the local microenvironment, creating a relatively hypoxic state favorable for bacterial persistence [27]. Regardless of whether the initial cause is intestinal barrier damage followed by bacteremia, or a primary local alteration of the thyroid microenvironment, the end result is structural disruption of the thyroid, which may create conditions for bacterial colonization and toxin production. Consistent with the proposed model described above, the thyroid may have contributed to systemic α-toxin exposure, potentially explaining the presence of toxin immunoreactivity in distant organs. Systemic exposure to α-toxin may be consistent with several necropsy findings in this case: α-toxin has dual phospholipase C and sphingomyelinase activities, directly disrupting cell membranes [28,29]. Severe intestinal tympany, hemorrhage, and extensive shedding of the gastric mucosa are consistent with the intestinal damaging effects of α-toxin [30,31]. Myocardial injury may have contributed to the large amount of pale-yellow pericardial effusion and auricular swelling/softening. Although primary cardiac disease cannot be completely excluded, no specific pathological evidence supported this possibility. Pulmonary hemorrhage is also consistent with the vascular endothelial-damaging effects of α-toxin [30,32]. It should be noted that blood cultures, blood PCR assays, and bacterial localization studies were not performed in this case; therefore, bacteremia and intra-thyroid bacterial invasion remain speculative. Therefore, the proposed “local proliferation–systemic intoxication” mechanism should be regarded as a hypothesis based on clinicopathological observations, not a proven pathogenic pathway.

We acknowledge that, as an isolated case report, this study cannot fully establish a causal relationship. Because this case involved a zoo giraffe, artificial infection experiments or animal model validation were not possible, and healthy giraffe thyroid tissue could not be obtained as a control. Therefore, establishing the above causal relationship has inherent limitations. Nevertheless, this finding has important value for the clinical prevention and control of Clostridium. It remains unclear what initially altered the thyroid microenvironment—for example, whether it was simple cold stress, feeding management issues, transient bacteremia, or a pre-existing unrecognized non-infectious thyroid lesion (such as goiter) in this giraffe. A major limitation of this study is the absence of direct evidence for bacteremia or hematogenous dissemination, and the bacteriological findings are based solely on the isolation of *C. perfringens* from thyroid tissue homogenates. In addition, the α-toxin antiserum used in this study was developed in-house. Although positive and negative controls supported its applicability, comprehensive validation in giraffe tissues was not performed; therefore, this remains another limitation of the study. Therefore, the proposed pathogenic mechanism should be regarded as hypothesis-generating rather than definitively established. Additionally, alternative explanations for the discordance between PCR and immunohistochemistry findings, including sampling effects, low bacterial burden, DNA degradation, postmortem redistribution, and differences in methodological sensitivity, cannot be completely excluded. Although the necropsy and tissue sampling of this case were performed within 1 h after death, which may have reduced the likelihood of substantial postmortem bacterial proliferation, postmortem contamination or bacterial overgrowth still cannot be completely excluded. Furthermore, blood samples were not obtained from this case for PCR detection of the *cpa* gene; thus, direct evidence of bacteremia is lacking.

This case highlights that, in animals presenting with clinical signs such as diarrhea, enteritis, or suspected bacteremia, especially under environmental stress, atypical infections associated with *C. perfringens* should be included in the differential diagnosis. Furthermore, when animals show unexplained neck swelling or signs of systemic infection, the possibility of *C. perfringens*-associated disease should be considered. In such cases, the thyroid gland may represent a potential site of bacterial colonization and should be included in pathological investigations when thyroid enlargement is observed [33]. In addition, this case suggests that husbandry management should be optimized during seasonal transitions, including improving thermal insulation, reducing stressors, appropriately regulating population density, and ensuring adequate individual nutrition, to minimize the risk of disease associated with opportunistic pathogenic bacteria [34]. In addition, the prevention and control of *C. perfringens*-associated diseases in captive animal populations depend on effective biosecurity measures, proper environmental sanitation, and routine health monitoring [35].

## 4. Conclusions

In summary, this study reports a rare case of acute thyroiditis associated with *Clostridium perfringens* type A in a giraffe, characterized by severe thyroid lesions and widespread α-toxin immunoreactivity. The pathological, PCR, and immunohistochemical findings are compatible with a scenario in which local thyroid colonization could contribute to systemic toxin exposure, although direct evidence of bacteremia or sustained toxin production is lacking. The thyroid may represent a potential site associated with atypical clostridial toxin exposure, and this case highlights the importance of considering systemic toxin effects in atypical clinical presentations. Future studies using controlled animal models and molecular markers are warranted to further explore the pathogenic mechanisms and clarify the role of thyroid involvement.

## Figures and Tables

**Table 1:** Detailed disease timeline from onset of diarrhea to death.

Day	Event
Day–5	Onset of diarrhea and weight loss
Day–4	Deworming administered
Day–1	Persistent diarrhea, no clinical improvement
Day 0 (morning)	Sudden abdominal distension
Day 0 (18:00)	Death

## Data Availability

The 16S rRNA gene sequence generated in this study has been deposited in GenBank under accession number PZ506140. Other data supporting the findings of this study are available from the corresponding author upon reasonable request.

## Supplementary Materials

The following supporting information can be downloaded at: https://www.mdpi.com/article/10.3390/ani16132006/s1, Figure S1. Specificity validation and cross-reactivity assessment of the homemade rabbit polyclonal antiserum against C. perfringens type A α-toxin by Western blotting. (A) M, protein molecular weight marker; lane 1 shows a specific immunoreactive band of the antiserum against purified α-toxin (~45.4 kDa); lane 2 shows C. perfringens ε-toxin, with no specific immunoreactive band observed. (B) M, protein molecular weight marker; lane 1 shows C. perfringens β-toxin, also with no specific immunoreactive band observed. These results indicate that the antiserum did not exhibit detectable cross-reactivity with the tested clostridial toxins (ε-toxin and β-toxin). Figure S2. Control staining for immunohistochemical detection of α-toxin. (A) Positive control: mouse intestinal tissue treated with α-toxin. (B) Negative control: mouse intestinal tissue without α-toxin treatment.
