# Supplementary material for: Fatal Acute Thyroiditis in a Giraffe (Giraffa camelopardalis) Associated with Clostridium perfringens Type A: A “Local Proliferation–Systemic Intoxication” Pathogenic Model
Source: Animals (Basel). 2026 Jul 1;16(13):2006. doi: 10.3390/ani16132006 (PMC13359913; doi:10.3390/ani16132006)
Supplement: Supplementary file 1 [file animals-16-02006-s001.zip › animals-4347628-supplementary.pdf]

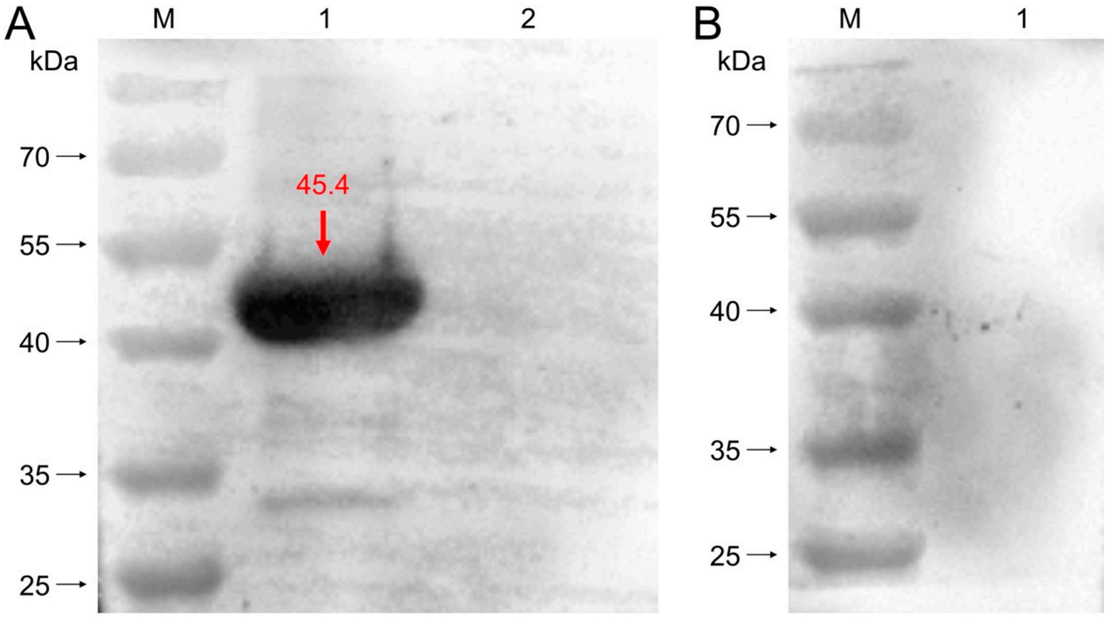

Supplementary Figure S1. Specificity validation and cross-reactivity assessment of the homemade rabbit polyclonal antiserum against C. perfringens type A α-toxin by Western blotting. (A) M, protein molecular weight marker; lane 1 shows a specific immunoreactive band of the antiserum against purified α-toxin (~45.4 kDa); lane 2 shows C. perfringens ε-toxin, with no specific immunoreactive band observed. (B) M, protein molecular weight marker; lane 1 shows C. perfringens β-toxin, also with no specific immunoreactive band observed. These results indicate that the antiserum did not exhibit detectable cross-reactivity with the tested clostridial toxins (ε-toxin and β-toxin).

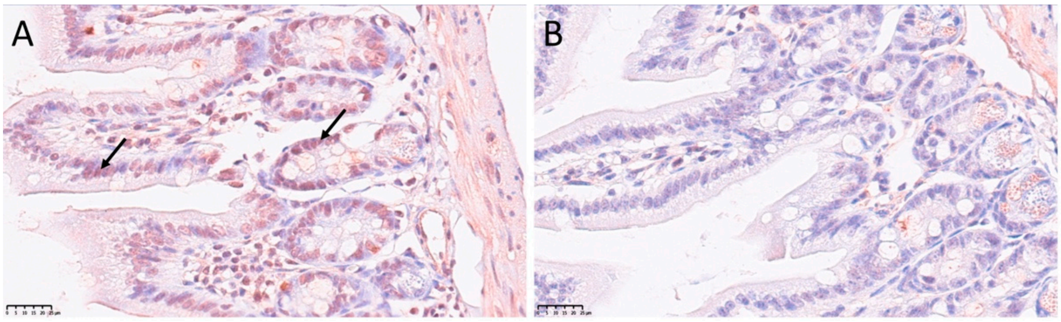

Supplementary Figure S2. Control staining for immunohistochemical detection of α-toxin.(A) Positive control: mouse intestinal tissue treated with α-toxin.(B) Negative control: mouse intestinal tissue without α-toxin treatment.
